# Respectability, morality and disgust in the night‐time economy: exploring reactions to ‘lap dance’ clubs in England and Wales

**DOI:** 10.1111/1467-954X.12278

**Published:** 2015-09-01

**Authors:** Phil Hubbard, Rachela Colosi

**Affiliations:** ^1^ University of Kent; ^2^ University of Lincoln

**Keywords:** class, gender, sexuality, urban sociology, sexual entertainment, night‐time economy

## Abstract

The night‐time economy is often described as repelling consumers fearful of the ‘undesirable Others’ imagined dominant within such time‐spaces. In this paper we explore this by describing attitudes towards, and reactions to, one particularly contentious site: the ‘lap dance’ club. Often targeted by campaigners in England and Wales as a source of criminality and anti‐sociality, in this paper we shift the focus from fear to disgust, and argue that Sexual Entertainment Venues (SEVs) are opposed on the basis of moral judgments that reflect distinctions of both class and gender. Drawing on documentary analysis, survey results and interview data collected during guided walks, we detail the concerns voiced by those anxious about the presence of lap dance or striptease clubs in their town or city, particularly the notion that they ‘lower the tone’ of particular streets or neighbourhoods. Our conclusion is that the opposition expressed to lap dance clubs is part of an attempt to police the boundaries of respectable masculinities and femininities, marginalizing the producers and consumers of sexual entertainment through ‘speech acts’ which identify such entertainment as unruly, vulgar and uncivilized. These findings are considered in the light of ongoing debates concerning the relations of morality, respectability and disgust.

## Introduction

Since the 1980s, there has been widespread concern that the more affluent and mobile classes have abandoned town centres in favour of out‐of‐town leisure and retail, leading to decline and even abandonment (Hubbard, 2003). Here it is worth remembering that the creation of leisured, 24‐hour cities was widely mooted in the 1990s as the solution to the problems befalling many town and city centres (see Oc and Tiesdell, 1997). Two decades on, such policies stand accused of rendering it more mono‐cultural by promoting a high‐volume ‘vertical’ drinking culture associated with working‐class excess (Hadfield, 2008; Nayak, 2006). Lurid images of binge drinking, anti‐social behaviour and disorder are widespread in representations of city and town centres at night, fuelling centrifugal tendencies in which older, wealthier and more mobile populations increasingly shun town centres because of fears of ‘undesirable Others’ (Eldridge and Roberts, 2008).

In this paper we explore the social conflicts that adhere to the contemporary night‐time economy by focusing on one particular site of contention: the ‘lap dance’ or striptease club.^1^ Increasingly prominent in England and Wales from the late 1990s (Colosi, 2010a), such clubs have widened the range of entertainment available to adult consumers. However, sexual entertainment also stands accused of coarsening the city, encouraging sexual immorality, and normalizing retrogressive attitudes towards women (Bindel, 2004; Jeffreys, 2008). In light of such opposition, the government passed clauses in the Policing and Crime Act 2009 granting local authorities in England and Wales the power to refuse licences for such venues in areas regarded as inappropriate (Hadfield and Measham, 2009). The subsequent licensing of such clubs as Sexual Entertainment Venues (hereafter, SEVs), and the continuing ‘backlash’ against them, has meant their numbers subsided from around 350 in 2004 to fewer than 215 dedicated clubs by the end of 2014.^2^ The closures have had sometimes devastating consequences for venue owners and employees alike (see Sanders and Hardy, 2012) but each is also heralded as a success in terms of improving community safety, and making women feel more comfortable in a night‐time economy ‘blighted’ by such premises (Patiniotis and Standing, 2012).

This paper accordingly explores the anxiety prompted by ‘lap dance’ clubs, drawing on an ESRC‐funded study of local residents’ attitudes, and emplaced reactions to, SEVs in England and Wales. This combined quantitative and qualitative methods, revealing that SEVs were not a major cause of distress to local residents, albeit a significant minority (of around one in ten) claimed to always avoid walking near such venues: women were significantly over‐represented in this group, suggesting the presence of sexual entertainment in the night‐time city does have important gendered effects. This was confirmed in guided walks conducted in four case study towns, which suggested women were more likely to note, and comment on, the presence of lap dance clubs than men (see Hubbard and Colosi, 2015). Yet in such instances, it appeared that unease about lap dance clubs was *more* related to questions of morality and disgust than fear, with SEVs’ contribution to criminal and antisocial behaviour deemed less significant by our participants than was the case for clubs, pubs or takeaways.

Our contention in this paper is that opposition to lap dance clubs is hence not about criminality *per se*, but needs to be understood in relation to particular assumptions that such sites act as a repository for unruly, debased and unmanageable identities and practices that threaten to ‘contaminate’ the wider town centre. Moreover, we show that this intersects with gendered norms to produce particular attitudes towards SEVs.^3^ In this paper we accordingly shift the focus of discussion around lap dance clubs from *fear* to *disgust*. Disgust is of course a contested concept in the social sciences, sometimes thought of as an ‘innate’ defence mechanism against contamination, other times as the product of ‘magical thinking’ which establishes impossible ideals of bodily perfection (eg Douglas, 1966; Rozin and Fallon, 1987; Sibley, 1995; Nussbaum, 2010). However, here we are concerned principally with the ‘cultural politics of emotion’ (Ahmed, 2004) and the deployment of language that sustains an emotional reaction of disgust towards particular bodies *and* spaces. Moreover, and following Tyler (2013: 21), we understand disgust to be an ‘urgent, guttural and aversive emotion’ which works, alongside other emotions, to create the ‘social distinctions of hierarchy and taste’ that infuse everyday life. Tyler suggests that disgust is frequently directed at the working classes, who have been repeatedly marginalized in Britain through popular culture's representation of them as vulgar and tasteless (see also Haylett, 2001; Lawler, 2005). In arguing that discourses of opposition to lap dance clubs serve to project negative values onto the ‘disrespectable’ working class Others assumed to provide, or consume, sexual entertainment, we extend Tyler's recent analysis of class relations in contemporary Britain by suggesting that the social disavowal, of sexual entertainment is implicated in the reproduction of, dominant notions of civility, morality and gendered respectability (see also Skeggs, 1997; Nayak, 2006).

## Exploring attitudes towards Sexual Entertainment Venues

Since the first opening of a dedicated SEV (*For Your Eyes Only*, in North London, 1996), there has been considerable public and political debate about such premises. Much of this has centred on questions of gendered exploitation, and related concerns about gendered violence. For example, Jeffreys (2009) contends that the striptease industry is a form of violence against women, reflecting the social misogyny deeply embedded in society. Others share Jeffreys’ sentiments: for example, Bindel (2004) depicts lap dancers as exploited women working in a patriarchal industry. Workers are described as pressured by male managers into taking on submissive ‘feminine’ roles for the benefit of (male) customer fantasies, leaving these women insecure about their physical and psychological identities (Wesely, 2003). Furthermore, it is suggested that dancers are frequently subject to verbal and physical abuse by both customers and managers (Holsopple, 1999). Other writers have highlighted the poor working conditions and limited employment rights of dancers (Sanders and Hardy, 2014). However, some accounts have countered by highlighting the complex power dynamics present in the club, suggesting that stripping can be empowering for some (see Colosi, 2010a, 2010b; Egan, 2006; Liepe Levinson, 2002; Sanders and Hardy, 2014). As well as being potentially lucrative, it is argued that lap dancers may find pleasure, and even fun, in their work through social interaction and improvisation (Colosi, 2010b; Sanders and Hardy, 2014). This deployment of pleasure and playfulness appears a way of resisting expected work practices, rules and regulations, with workers deriving a sense of empowerment through forms of ‘anti’‐work (Colosi, 2010b).

In relation to criminality, there has been a significant debate as to whether the presence of clubs is associated with increased rates of both violent and non‐violent crime. While most academic studies emanating from the US demonstrate little association between crime and the presence of ‘adult premises’ (eg Linz *et al*., 2000; Paul *et al*., 2001; Hanna, 2005; McCord and Tewksbury, 2012), the lack of statistical evidence of criminality may not of course tell the full story. As opponents of sex premises assert, fear of crime can be as significant as crime itself, with the presence of venues creating ‘no go’ areas – particularly for women. For example, Patiniotis and Standing (2012) conducted a small‐scale project exploring local women's perceptions of SEVs in the north‐west of England. Here, participants were asked to take pictures of spaces where they felt disempowered or empowered, leading to the conclusion:

It is the existence of the clubs that causes women to feel alienated in public spaces at all times, and fearful of the threat of violence posed by the sexual objectification of women on display outside the clubs and acted out within them. (Patiniotis and Standing, 2012: 11)

This argument has featured prominently in campaigns against lap dance clubs, particularly the ‘Stripping the Illusion’ campaign led by feminist group *Object* (see Eden, 2007). However, despite such claims about fears of crime around SEVs, there have been no academic studies gauging perceptions of lap dance clubs across a wider range of local residents, both female and male.

In this light, and with an eye to informing policy debates concerning the licensing of SEVs (lap dance clubs), we performed a study of attitudes to such premises. The project examined local residents’ perceptions of SEVs in four case study locations across England and Wales. All four locations had all adopted the new licensing powers as outlined in section 27 of the Policing and Crime Act 2009, and were selected because they included a range of different styles and settings of clubs. Location one was a small county town with little history of sexual entertainment containing one SEV; location two was a mid‐sized city boasting two SEVs. The third location had a more substantial night‐time economy (including several venues marketed at LGBT consumers) and three SEVs. Finally, location four was a provincial city known for ‘stag and hen’ tourism, hosting four SEVs and several premises that offer striptease entertainment on football match days.

In the first instance, we completed a discourse analysis of the formal objections made to the SEVs licensed in each location up to 1 April 2012 (ie in the first year that licences could be applied for). These objections were often few in number – and several clubs were not objected to at all – but the fact they had been solicited through well‐advertised processes allowing interested parties to express opinions about the appropriateness of specific venues meant they were revealing of concerns that might have been more widely shared. To explore this, residents in each of the four locations were recruited to complete an online survey that aimed to explore attitudes to SEVs. In total 941 residents completed this survey, which prompted for knowledge of, attitudes towards and perceptions of SEVs in their local towns. This survey also prompted for basic socio‐economic characteristics to allow for comparison of findings by age, gender and race/ethnicity: to ensure the surveys were broadly representative of local populations, flyers inviting participation were hand‐posted to households in three contrasting neighbourhoods in each case study location.

Following analysis of the survey data, 46 respondents were recruited from the survey to participate in eight evening guided walks, with participants being initially selected to represent a diversity of age and gender positions. These guided walks took place in all four locations, with five to eight respondents in each event. This approach helped provide us with more qualitative material relating to the respondents’ feelings about different parts of urban centres, with routes chosen to ensure all of the SEVs would be visible at some point in the walk. These guided walks lasted between one and two hours, with participants incentivized to participate through shopping vouchers. During these walks, we found ourselves searching for clues from the body language of participants, reflecting on where they were comfortable stopping and talking, and where they wished to leave, as well as listening to what they told us as we walked around the case study towns. All walks were digitally recorded and transcribed, with a visual record collected by asking respondents to indicate anything they would wish us to photograph as indicative of the night‐time economy (see O'Neill and Hubbard, 2010, on guided walks as sociological method).

Our methods were hence based around a specific set of prompts asking respondents to state their opinions about venues in specific locales. Rather than exploring abstract or generic attitudes towards sexual entertainment, this project aimed to explore the emotions evoked by proximity to, and the sight of, lap dancing venues *in situ*. What emerged from this analysis was that the dominant emotional response appeared to be one of disgust rather than pain, hate, fear, or shame. This is not to say that occasionally emotions of shame or anger did not emerge, but that disgust and repulsion appeared predominant.

In alighting on disgust – rather than fear, hate, or desire – as the dominant emotion expressed towards lap dance clubs, we are making a distinction between disgust as an atavistic and primal reaction to that which literally endangers us, and the feelings of disgust projected upon those we are repeatedly told are a danger. As such, we follow Nussbaum (2010: 15) in distinguishing between the ‘primary objects of disgust’ which ‘are the reminders of human animality and mortality: feces, other bodily fluids, corpses, and animals or insects who have related properties (slimy, smelly, oozy)’ and the manipulation of this disgust by dominant groups seeking to cordon themselves from their own animality. Nussbaum (2010) considers nudity on several occasions, arguing that the sight of the naked body is widely accepted to cause disgust when encountered in public, despite no clear sense of what harms this causes the adult viewer. Indeed, in some contexts a naked body may be considered as artful or beautiful – an object of desire – rather than obscene or immoral (Nead, 2002). Nussbaum nonetheless argues that a legitimate reason to ban public nudity is the concern that it threatens the civility of public space. Here, she notes there is no particular agreement about what the harms caused by the naked body might be, yet argues that banning public nudity is legally reasonable on the basis that there is a social consensus that those who expose themselves, or are exposed, are disgusting. The increasing sexualization of the naked body, and its equation with danger, has thus served to undermine other possible discursive framings of public sexuality, such as those related to health and freedom (Barcan, 2004). This is important in the context of our argument given sexuality is a realm where the social worth and meaning of different identities, bodies and subjectivities remains fiercely contested (McRobbie, 2007).

Given the evident lack of consensus about the harms of nudity, the social taboo around public nudity appears as much connected to notions of social refinement and taste as to fears of contamination per se (see also Cooper, 2011). This said, given the nakedness of the striptease artiste in a lap dance club is rendered invisible to all but paying (adult) customers, the disgust prompted by the presence of the club is not so much a reaction to the primary object of disgust, but the projection of moral disgust upon those associated with it. This disgust appears prompted by the disavowal of respectable standards of sexual behaviour and comportment, not nudity per se. Drawing on discourses of opposition and the anxiety expressed towards clubs, we accordingly argue that the male customers of such venues are regarded as embodying an unruly (working‐class) masculinity, and the female performers viewed as failing to uphold notions of respectable (middle‐class) femininity. Both good taste and morals are considered to have been violated, with the consequence being a specific linking of the individual and the social: those expressing disgust are constituted as respectable – yet ‘anxious and defensive’ – subjects (Lawler, 2005: 443).

In the remainder of this paper we use our data to highlight three prominent discourses that revealed, and sustained, the emotional reaction of disgust expressed towards these venues. The first concerns the idea that the venues in which performances occur do not belong in ‘respectable’ communities; the second is that their male clientele represent a dangerous residual masculine culture; and the third that the women who perform in these clubs embody non‐aspirational values that do not accord with dominant notions of feminine respectability. Our analysis accordingly suggests that there are important differences in the framing of clients and dancers, with the women who perform in lap dance clubs figures of pity as much as figures of disgust. This negative framing, however, still fuelled the disgust reactions registered by many of our participants who positioned lap dancers as the disrespectable Other. In conclusion we suggest that these three ‘speech acts’ work together to reaffirm the boundaries of respectability, while denying access to this identity for those proclaiming lap dance clubs to have a legitimate place in the night‐time economy. Here, we are not so interested in describing variation in attitude across boundaries of class, gender or age, merely demonstrating that a *shared* language existed which reproduces dominant social hierarchies of respectability, worth and morality. As such, we conceptualize disgust performatively both as ‘the intensification of contact between bodies and objects’ and ‘also as speech act’ (Ahmed, 2004: 92) which creates and sustains social distinction.

## Vibrant clubs or a vulgar presence?

The most frequently voiced assertion in public objections to SEVs is that they constitute an inappropriate presence in particular locales. Clubs are routinely described in submissions to licensing committees as ‘lowering the tone’ of particular areas, or constituting an ‘insalubrious’ presence. This is an argument that is made contextually: some clubs are deemed to be ‘wholly inappropriate’ in certain areas by virtue of the proximity of other land uses imagined to be vulnerable or ‘sensitive’. Here, the common suggestion made is that location of important amenities in the immediate vicinity of premises would directly expose users of those amenities to specific harms and/or externalities. While places of worship are mentioned in a number of objections, the overwhelming emphasis is the incompatibility of lap‐dancing clubs with facilities associated with children: as one objector put it, ‘having venues for sexual entertainment within the sight of popular family areas is a disgrace’. Overall, our survey revealed that 83 per cent of people think that SEVs are unsuitable near schools or nurseries, 46 per cent near universities/colleges, 65 per cent near religious facilities, and 45 per cent near shops. Likewise, only 3 per cent think such clubs are suitable in residential areas, 10 per cent in rural areas, and 15 per cent in industrial areas, though 55 per cent felt town centres are suitable in general terms. The latter implies an imagined geography of class and morality where sexual entertainment does not belong in suburbia or the rural, but can be seen to belong only in the town centre or inner city (areas that have become associated with what Tyler, 2013, describes as a torrent of ‘underclass’ appellations). This helps construct what Hubbard (2011: 34) refers to as a ‘moral geography’ in which there are clear assumptions about what behaviour belongs where: this is mirrored in legislation which seeks to steer clubs away from ‘inappropriate’ locations, inevitably pushing it towards less valued neighbourhoods (see also Edwards, 2010).

Yet the idea that clubs belong at all in night‐time economies was disputed by some – around one in ten – who felt that such clubs always represent a vulgar presence:

Well I have to say I hate the thought of having one here. I've never heard any rowdy behaviour or anything … But I don't like the thought of lap dancing, I think that it's just tacky. (Guided walk participant, female, 40s)

More widespread, however, was directed opposition suggesting that the proliferation of sexual entertainment would precipitate a process of local decline:

The tone of the neighbourhood will be lowered, residents will think twice before leaving their property in the evening. There will be reduction in property value – people will not want to live near such an undesirable business. (Objection to a club included in licensing committee agenda papers, 24 May 2011)

This area has historically suffered from a stigmatised image and efforts have been made to regenerate the area to benefit businesses and residents alike. The presence of a sex industry premise confounds this image and undermines such efforts. (Objection to a club included in licensing sub‐committee agenda papers, 19 July 2011)

Hence, although concerns were aired about the impact of clubs on local crime rates, by far more numerous were discourses describing these venues as ‘tasteless’ or ‘vulgar’, with the survey confirming 60 per cent believe the presence of an SEV damages the reputation of an area, double the proportion that feels it reduces safety on the street.

It is important here to reflect on how disgust sustains or preserves the connection between ideas (eg vulgarity), values (eg respectability), and spaces (eg the lap dance club). Following Ahmed (2004), a consideration of the transference of emotion is important: disgust here is not prompted by an encounter with the performance of striptease per se, but the sight of the venue. The overt representation of sexuality through club signage, names and flyers (such as those containing images of semi‐clad females in provocative poses) appeared highly significant in this respect. Indeed, respondents expressed a shared opinion that venues should be ‘low key’ and inconspicuous. Those with children justified this in terms of having to explain to them the nature of the entertainment which takes place inside if asked. One of the respondents relayed her experience thus:

I can remember my son asking ‘what's that then?’… having a conversation with my husband and I was just thinking how do you explain that to a six year old who's completely innocent? I don't know, since I've had children it bothers me but I think before I wouldn't have given it much thought. (Guided walk participant, female, 40s)

So while the obvious materialization of sexual entertainment in the cityscape does not prefigure particular affects, the sexualization of space appears to create an atmosphere that Ahmed (2007: 125) suggests will always ‘angle’ the way that a situation or space is encountered, and prompt responses that are informed by dominant social and cultural norms.

Here, a key theme that emerged from our roving interviews was the sense that visibly advertised SEVs shifted the ‘metabolism of the city’ (Cronin, 2006) by sexualizing particular streets at particular times. It has been argued elsewhere that sexualized advertisements make women feel uneasy on a number of levels, with the display of pseudo‐pornographic pictures and women in a state of semi‐nudity constituting a form of *sexual harassment* (Rosewarne, 2002), something around which guidelines have developed (such as those developed by the Advertising Standards Authority in the UK). It was noted by several of our respondents that gendered images and representations were embedded in urban space through club's flyers, advertising boards and signage: while local authorities demand that advertising is discreet and non‐explicit, the names of clubs (eg *Beavers, The Pussycat Club*) are sometimes far from subtle, with ever‐present images of sexualized bodies on billboards, signage and advertising hoardings effectively reminding viewers that the city is a sexual marketplace where bodies are constantly on display and all is for sale, with the female form being used to *seduce* the viewer:

The advertising material consists of a display board with colourful writing advertising exotic dancers with the image of a silhouette of a pole dancer This type of material is highly visible to any tourist or visitor … the promotion of these activities makes the High Street seem seedy and uninviting. (Objection to a club included in licensing committee agenda papers, 23 October 2011)

Local authorities routinely demand that no part of the interior of a club is visible from the outside, meaning the nature of the entertainment is concealed from passers‐by. Yet the idea that the appearance of a club can make an area feel threatening was played up by several participants in our walk along event, including one who suggested that a club contributed to ‘a sleazy atmosphere with its blacked out windows’ (guided walk participant, female, 40s) and another who argued that a club ‘just looks sinister’ (guided walk participant, male, 30s). This suggests that clubs can effectively sexualize the cityscape via an aesthetics of concealment and seduction which our respondents often claimed to be sleazy and vulgar.

Disgust here was an emotion that appeared most strongly expressed when in proximity to a club, underlining the assertion that affective experience is a cumulative, historical, process of interaction between people and place that can lead to a strong sense of the abject (Ahmed, 2004). In this sense, notions of class, immorality and deviance were connected via the materialization of lap dance clubs in the landscape, leading to in situ reactions based on notions of disgust (see also Sibley, 1995). This implies that while most claim they are not opposed to sexual entertainment venues in general terms, when such clubs become visible in particular settings they can arouse widely shared concerns and anxieties (cf Hubbard *et al*., 2013). This geographically informed perspective on disgust suggests that the interpretation of respectable standards of behaviour and comportment is something that needs to be understood relationally and contextually, with the transgression of ‘civilized’ standards of comportment mapped onto specific places and objects.

## Gentlemen or ‘unsavoury’ males?

Given the importance of proximity as something that can provoke disgust reactions, it was interesting that 22 per cent of those who lived in a town or city with an SEV were unaware that any club was present. Of the remainder, most had become aware of a venue by seeing it on the street rather than reading about it in the media, though one in four of those who were aware of such premises had visited a venue: interestingly, this included similar proportions of men and women. Yet irrespective of whether someone had visited a club before or not, a common trait was to speak of the clientele of clubs, in blanket terms, as a working class ‘Other’ (see Tyler, 2013). Again, drawing upon Ahmed's (2004) ideas, it appears it is the lap dance club that is seen to be contaminating those who are associated with it – in this instance the clientele – who are then too considered disgusting. Here, the clientele were uniformly depicted as unruly and dangerous, embodying unrespectable working‐class masculinities (Tomsen, 1997), with our respondents appearing to locate themselves within middle‐class frames of comportment by using particular language. Interesting here was the repeated assertion that the portrayal of ‘lap dance’ venues as ‘gentleman's clubs’ was a complete misnomer:

No gentlemen would frequent a bar like this. I think if he was to say: ‘no I'm not going to frequent such things that are degrading’ – I would maybe think of him more of a gentleman. (Guided walk participant, Female, 20s)

In a letter opposing licensing of an SEV, one objector rhetorically asked ‘Do you want [our city] to return to caveman's times?’ (letter included in licensing committee agenda papers, 27 April 2012). This type of moral judgement identifies the clients of lap dance establishments as failing to exercise civilized standards of comportment, with the (assumed male) client described in pejorative terms:

I don't know what it is about that club but it seems to attract a lot of unemployed people that are off their heads and you get them sitting outside, on these benches, looking really out of it and it's really off‐putting. I don't know why they are all in that one and they don't go anywhere near this one. They changed the name and revamped it but it's still got the same reputation as it has before (Guided walk participant, female, 30s).

Likewise, according to one resident opposing the licensing of a club: ‘they are seedy and unpleasant individuals’ (objection to a club included in licensing committee agenda papers, 2 May 2012). Another argues clients are ‘A certain calibre of person, attracting lurid behaviour, drink and drugs’ (objection to a club included in licensing committee agenda papers, 24 June 2011).

The language used here echoes the hate speech discussed by Tyler (2013) when she talks of the ‘scum semiotics’ used to portray the British ‘underclass’ by repeatedly reinforcing notions that there is a social hierarchy in which the consumption of sexual entertainment is associated with a ‘sexual underclass’. The suggestion here is that those claiming to belong to the ‘respectable classes’ do this by complaining about, and dissociating themselves from, the excessive and vulgar behaviour they associate with the faction of the working classes that is disrespectful and ill‐mannered (Tomsen, 1997). Inevitably, this is mapped onto space, and finds in expression in NIMBY (‘Not in My Back Yard’) protests against ‘sex premises’ (Hubbard *et al*., 2013): our respondents hence frequently spoke of customers as failing to recognize local norms of behaviour, and disturbing community ways of life:

The club is a venue for the binge‐drinkers who is [*sic*] seeking drugs and prostitution: their loud behaviour, screaming, shouting, swearing, running echoes round the streets. Empty bottles, broken glass vomit and urine can be seen around the pavement in this area. (Guided walk participant, female, 30s).

This chimes with the allegations made in letters opposing the licensing of sexual entertainment venues, where the behaviour of clients is often described in similar terms:

Our street serves as the car park for the groups of often drunk, loud and obscene punters who roll up, clearly seeing a visit to a lap‐dancing club as the end of a glorious night's drinking. These men travel in packs, and engage in displays of bravado, frequently swearing, urinating in gardens and egging each other on … Nobody in our neighbourhood can take a walk without risking encounters with the clients. (Objection to a club included in licensing committee agenda papers, September 2011).

The deployment of animalistic metaphors (ie travelling in packs) hints at the projection of non‐human traits onto stigmatized groups (Rozin and Fallon, 1987; Sibley, 1995), and constructs a moral hierarchy in which those who purchase sexual entertainment are ‘dehumanized, dirty and animalistic’ (Campbell and Storr, 2001: 98). As Kingston (2013: 17) notes, there is often little difference in the language describing those who buy sex and those who consume sexual entertainment: both are portrayed as a ‘predator who needs to be guarded against, external to the community of “normal” men – an outsider who is untrustworthy and “dangerous”’. As one of the participants in a guided walk commented: ‘in the club the local women are yours for the taking if you want and then they [men] have a drink, and this then progresses onto the streets, and when they're in their packs and if you happen to be there … who knows what’ (guided walk participant, female, 30s).

Here, there is some degree of conflation between the clientele of the lap dance club and a stereotyped ‘lager lout’ who is commonly depicted as ‘literally spilling out of one drinking den into the next one and, all the while, representing the potential for disorder and the transgression of propriety (Thurnell‐Read, 2013). This figure is clearly classed and gendered, seen to disdain respectable masculinities (Skeggs, 1997; Tyler, 2008). Rather than keeping ‘immoral’ behaviour inside the club, clients were described as taking this onto the surrounding streets, ‘polluting’ moral order:

I don't like somewhere like here. Because of the people who visit a place like this. The behaviour you'd expect from people who visit places like this, directed at women who are not associated with that but they don't seem to distinguish between women who might be in there and women on the street. It sort of like spills out and affects the surrounding population if that makes sense? (Guided walk participant, male, 50s)

Such speech acts again emphasize the ‘stickiness’ of affect (Ahmed, 2004), and the importance of disgust as an emotion that occurs in response to the proximity of contagion (whether real or imagined). Of course, such speech acts betray ‘magical thinking’ about the connections between moral and physical contagion (Nussbaum, 2010): moral disgust is, it can be argued, ‘normatively irrational’ and does not help us identify what – or whom – is truly dangerous (see Sibley, 1995).

This said, multiple representations against lap dance clubs allude to possibility that ‘patrons who are generally intoxicated and arguably in a state of sexual excitement’ might harass local women, noting that venues often target ‘stag parties which are renowned for their negative impact in relation to crime and disorder’ (objection to a club included in licensing committee agenda papers, May 2011). For instance, one of our participants during a guided walk expressed her anxiety about the effect upon the men visiting these venues, suggesting that she believed they put female members of the public at risk: ‘…it's just feeding them [men] frenzy, and then they go out on the street and we [women] get all the shit’ (guided walk participant, female, 30s). However, the same respondent also stressed that she had not actually witnessed or personally experienced any threatening behaviour whilst in close proximity of an SEV, suggesting that disgust may lead to other affects – such as fear – rather than vice versa. Indeed, allegations of serious sexual assaults perpetrated by clients against women are extremely rare, with the most frequent accusations being simply that clubs attract ‘unsavoury’ male visitors, thus making the areas around clubs feel uncomfortable for female passers‐by (Hubbard and Colosi, 2013).

## Meaningful employment for women or ‘dirty work’?

If the male clients of striptease prompt disgust because their behaviour seems to embody uncivilized values, how are female performers perceived and understood? Our results suggest women working as lap dancers still prompt negative emotional reactions, albeit these are couched in terms of *pity* as much as disgust. This relates closely to the assumption made that lap dancing itself is disgusting and does not constitute respectable work. Therefore the pity directed at lap dancers positions these women as the disrespectable Other, placing them in opposition to dominant notions of respectable femininity. The notion that striptease is not respectable and instead positioned as dirty, demeaning and exploitative, is one that seems to have permeated public discourse, even though some dancers and ex‐dancers have stressed lap dancing can be empowering or even emancipatory (see Liepe Levinson, 2002; Egan, 2006; Colosi, 2010b). A subtext here is that women do not become lap dancers out of rational choice, but instead do so as a result of coercion or ignorance (Colosi, 2010a). For example, one of our respondents argued that lap dancing is a job borne of necessity: ‘they are doing it as a means to an end … it's not what they want to do’ (guided walk participant, male, 40s). This is, however, contrary to some of the accounts given by lap dancers who claim that they engage with this work in pursuit of fun and enjoyment (Colosi, 2010b; Sanders and Hardy, 2014).

A frequent argument spelt out in objections to new clubs in our case study towns was that owners, managers and customers would pressurize dancers into providing sexual services. For example, objecting to the licensing of clubs in one of the case study locations, multiple residents referred to a study that

Some lap‐dance club owners and managers create a context in which the buying and selling of sexual services may occur … club owners tend to absolve themselves of any responsibility if sexual services are found to be occurring or being arranged on the premises, yet at the same time there is some indication that they encourage the dancers to project an air of sexual availability to customers. By making it difficult for the dancers to earn an adequate living legitimately, through requiring the payment of ‘rent’ for each shift worked in the clubs, and by hiring excess numbers of dancers at any one time, club owners and managers also create a series of structural conditions that can lead some dancers to offer sexual services in order to survive financially. (Bindel, 2004: 59)

The idea that performing striptease is demeaning to performers, and that the women who are employed in such venues are encouraged to offer sexual services was a prominent discourse amongst our participants. Such assumptions of coercion and exploitation were frequently voiced, leading to the occasional description of clubs by our respondents as ‘little better than brothels’.

The association made between prostitution and lap dancing was further reflected in some of the views expressed by residents on our guided walks, with one resident bluntly asserting: ‘I feel that lap dancing is a form of prostitution’ (guided walk participant, female, 30s). The association made between prostitution and lap dancing emphasizes the forms of disgust directed at lap dancing, with the distinctions made between ‘clean’ and ‘dirty’ work, positioning lap dancing, and the women who work in these clubs, as ‘dirty’ (Mavin and Grandy, 2011). Furthermore, irrespective of the skills employed, sexual entertainment appears to be a form of work that cannot be performed in a way that allows a ‘bad girl’ access to the category of ‘good girl’ or worker:

the nature of sex work is such that it symbolizes bad sex (sex outside marriage, public, promiscuous and not for reproductive purposes). So while being good at their work through exaggerated expressions of doing gender (and thus good workers), sex workers are still perceived to be bad or dirty workers. (Mavin and Grandy, 2011: 232)

This association of dirt and disgust was underlined in discourses which positioned the lap dancer as ‘victim’, with our respondents frequently emphasizing the exploitative nature of this work, and one female guided walk respondent in her 40s making reference to ‘sex trafficking’. This perhaps reflects wider political and media discourses which attempt to make strong links between the sex industry and trafficking, despite limited evidence to support this assertion (Weitzer, 2007). In identifying lap dancing as intrinsically exploitative, one of our respondents on the guided walks argued this is unavoidable, given the nature of the work, relating it to the (imbalanced) power dynamics between lap dancer and client:

I think it's who's got the money and who's got the control. And there's [*sic*] loads of issues around that … So I do think it's a lot of stuff around power and things like that… (Female respondent, 40s)

The issue of exploitation was further emphasized by another respondent, who questioned the notion of ‘empowerment’ in the lap dance club environment:

I mean my feeling is that it's disempowering for women. And this curious argument that this is empowering for women … and the argument that the lap dancers give that they feel empowered and [feel] so much better than the men, and I was like well how's that benefitting society in a wider way? If you feel you are better than the person that's essentially paying your wages? (Guided walk participant, female, 20s).

Not only did this respondent identify lap dancing as disempowering, she questioned the value of lap dancers’ labour, and in doing so, suggests those working in this industry have poor aspirations. Here, the disgust directed at sexual entertainment is again emphasized, further distinguishing lap dancing from ‘respectable’ (and ‘clean’) forms of work.

The idea that striptease is a form of labour different from any other is something which was hence stressed by respondents through a language which mirrors Jeffreys’ (2008: 161) assertion that ‘there is no other form of work, except in the sex industry, where women have to battle to keep their bodies away from men's fingers and ejaculate’. The idea that this is a job involving the ‘lower regions of the body’ (Stallybrass and White, 1986: 9), rather than one involving skills of negotiation, interaction and emotional management (see Colosi, 2010a), is then important in figuring the lap dancer as on the constitutive limit for clean, middle‐class femininity. As one male participant boldly stated, ‘I wouldn't touch them with a barge pole’ (guided walk participant, male, 40s). The pity invoked in respondents’ accounts appeared suggestive of a (middle) classed position which situates sexual entertainment, and those associated with it, as disrespectable:

The women themselves … scrape a living working in these clubs. Not only are there many issues around personal safety but also mental well‐being if those who are routinely degraded for a living by men who see them as sex objects … many women who end up working in these environments have previous experiences of sexual abuse – how cruel they now accept this as a norm in their working lives in a ‘legitimate’ licensed premise. (Objection to a club included in licensing committee agenda papers, 26 June 2011).

Denying the possibility that lap dancing can be financially rewarding, describing this as an illegitimate business cements the association between criminality and striptease entertainment that is prevalent in media and public discourse: this is an association which helps to further identify the lap dancer as failing to perform a respectable class identity.

As Skeggs (2004: 24) argues, working‐class women struggle to ‘truly inhabit the category of femininity’, with middle‐class disgust for the sexualities of working‐class women a frequently noted trope (see also Tyler, 2008). Evidentially, lap dancers also find it impossible to conform to working‐class ideals of respectable femininity, and appear to represent a ‘failing femininity’ (Allen, 2014: 765). In a society where womanhood tends to be defined around middle‐class and upper‐class ideals, the hypersexualized performance of the lap dancer can be dismissed as what Skeggs (2001: 298), drawing on the work of Carole Anne Tyler (2003), refers to as a ‘class drag act’.

Here it is important to note that while our guided walks around towns with SEVs present included women recruited from a variety of backgrounds, some of whom evidentially knew people who worked in the clubs in question, the format of a group conversation led to a consensus of opinion that clubs were exploitative of women, and that aspiring to work in one of them was not appropriate. This of course may partly be a function of our method, given participants on guided walks were accompanied by middle‐class academic researchers. Nevertheless, all our participants gave themselves value and authorized their opinions by articulating the argument that this form of employment was demeaning and disgusting (cf. Skeggs, 2004).

## Conclusion

It has been widely argued that town and city centres at night are ‘inherently dangerous environments’ associated with uncivilized, disorderly and excessive behaviour. Sexual entertainment appears to have played a role in heightening such perceptions, as it appears to embody a vulgarity and immorality that generates disgust when encountered in the cityscape. In this study, we found that those providing sexual entertainment were routinely represented through a language of ‘social abjection’ (Tyler, 2013) that combined condemnation and pity, while there was a more overt demonization of the male consumers of sexual entertainment. In turn, spaces of lap dancing were deemed potentially contaminating, their presence condemned. Here, it appears that speech acts describing such premises as ‘disgusting’ or ‘immoral’ may be important in creating a community bound together ‘through the shared condemnation of a disgusting object or event’ (Ahmed, 2004: 94).

In this sense, our analysis suggests that the expression of disgust towards sexual entertainment is one means by which it is possible for an individual to claim respectability, disavowing any public identification with forms of behaviour that have become depicted as vulgar and uncivilized (Tyler, 2013). Distinction is hence expressed through a language of disgust in which the behaviour of the Other is used to demonstrate the Self as cultured and civilized. As such, it appears impossible to reproduce anything other than a classed discourse about lap dance clubs – even if this was vested in a language which was more about femininity and respectability than outright condemnation of working‐class identities (see also Skeggs, 1997, on dis‐identification). Those who felt sexual entertainment was harmless, good fun, or a viable form of employment for women certainly did not claim this when participating in our guided walk event, albeit online survey data revealed a more ambivalent attitude. It appears that to publicly admit to enjoying, or even tolerating, sexual entertainment would be to admit a lack of taste, identifying one as devoid of value, and immoral (Lawler, 2005). Our analysis accordingly infers that the opposition expressed to lap dance clubs can be interpreted as part of an attempt to police the boundaries of middle‐class respectability, Othering the producers and consumers of sexual entertainment through ‘speech acts’ which identify such entertainment as unruly and vulgar. Indeed, the language used to describe the male clientele of lap dance clubs speaks to wider concerns about masculinity and class (Thurnell‐Read, 2013), while the disgust registered about women selling sexualized performance echo long‐standing stereotypes of working‐class women as wanton, immoral and exploited (Mavin and Grandy, 2011).

This paper has shifted the debate around lap dance clubs from being solely about (gendered) fear to one about respectability pity and disgust. By examining how such clubs, along with the producers and consumers of sexual entertainment, evoke emotional reactions, we have sought to highlight opposition to the lap dance club that positions these venues as repositories of unruly masculinities and sites where respectable femininity is compromised. Turning such conclusions around, we can begin to sense some of what Tyler (2013) talks of when she refers to the continuing devaluation of working‐class cultures created through repeated disgust reactions. As she notes, it is through association with abject sexuality that working‐class women and men are frequently stigmatized and disavowed. The emergence of a dominant way of speaking about ‘lap dance’ clubs suggests that repetition of such discourses does more than simply stigmatize lap dancing: it also circumscribes moral subject‐positions and reproduces dominant notions of gendered and classed respectability.

## Notes

1 The description of premises offering striptease performance as ‘lap dance’ clubs is a misnomer given local authorities typically ban any contact between dancer and customers. Many of the clubs are actually marketed as ‘Gentleman's clubs’: however, we use the terms ‘lap dance club’ and ‘striptease club’ interchangeably in this paper given this is the way they are typically described in popular and public debate.

2 The majority of these offer female striptease performance to an (assumed male, heterosexual) audience, though a number occasionally host male dancers for female clientele. As of 2014, there were also four ‘gay’ clubs licensed as Sexual Entertainment Venues, and only one ‘lesbian’ club licensed in this way, all located in London.

3 It needs to be recognized that these classed judgements can intersect with other identity markers such as ethnicity. However, while there are media discourses which highlight the ‘exotic’ Otherness of those who work in sexual entertainment, here we focus on the intersection of class and gender as ethnicity was not invoked by any participants in our research, and did not appear to be a significant factor in the disgust reactions registered in these case study locations.
